# A Thermostable β-Glucuronidase Obtained by Directed Evolution as a Reporter Gene in Transgenic Plants

**DOI:** 10.1371/journal.pone.0026773

**Published:** 2011-11-09

**Authors:** Ai-Sheng Xiong, Ri-He Peng, Jing Zhuang, Jian-Min Chen, Bin Zhang, Jian Zhang, Quan-Hong Yao

**Affiliations:** 1 State Key Laboratory of Crop Genetics and Germplasm Enhancement, College of Horticulture, Nanjing Agricultural University, Nanjing, China; 2 Biotechnology Research Institute, Shanghai Academy of Agricultural Sciences, Shanghai, China; 3 Alberta Innovates-Technology Futures, Vegreville, Alberta, Canada; 4 College of Bioscience and Biotechnology, Yangzhou University, Yangzhou, China; National Institute for Medical Research, Medical Research Council, London, United Kingdom

## Abstract

A β-glucuronidase variant, GUS-TR3337, that was obtained by directed evolution exhibited higher thermostability than the wild-type enzyme, GUS-WT. In this study, the utility of GUS-TR337 as an improved reporter was evaluated. The corresponding *gus-tr3337* and *gus-wt* genes were independently cloned in a plant expression vector and introduced into *Arabidopsis thaliana*. With 4-MUG as a substrate, plants containing the *gus-wt* gene showed no detectable β-glucuronidase activity after exposure to 60°C for 10 min, while those hosting the *gus-tr3337* gene retained 70% or 50% activity after exposure to 80°C for 10 min or 30 min, respectively. Similarly, *in vivo* β-glucuronidase activity could be demonstrated by using X-GLUC as a substrate in transgenic *Arabidopsis* plants hosting the *gus-tr3337* gene that were exposed to 80°C for up to 30 min. Thus, the thermostability of GUS-TR3337 can be exploited to distinguish between endogenous and transgenic β-glucuronidase activity, which is a welcome improvement in its use as a reporter.

## Introduction

There are about fifty reporter genes that have proved useful in transgenic plant research, based on their efficiency, scientific applications and commercialization. Reporter genes serve as indicators to study transgenic events, for example by facilitating visual identification of successfully transformed entities in a large background of non-transformed material. Although many reporter genes have been proposed, only a few of them have been commercialized [1]. β-glucuronidase (GUS) [2], [3], green fluorescent protein (GFP) [4], [5], luciferase (LUC) [6], [7], [8] are three important reporters that have a proven track record in transgenic plant research.

The advantage of GFP and LUC as reporters includes the possibility of monitoring in live tissues and in real time [1], [4], [8]. These two reporters allow continuous monitoring of the gene activity even through the developmental stages of the transgenic plants [1], [5], [9]. However, there are several disadvantages in the use of these two reporter genes. For example, the half life of LUC is short (a few hours) in plants and the process of catalyzing the release of oxyluciferin is slow [6], [7], [10]. In case of GFP, there may be interference due to a high background of auto-fluorescence. Another major drawback in the use GFP as a reporter is that its detection requires relatively expensive instrumentation, namely the fluorescence microscope.

The bacterial enzyme GUS encoded by the *E. coli gusA* gene is to date the most widely used as a reliable transgenic reporter system in transgenic plant research [1]. GUS has some advantages as a reporter in transgenic plant research and commercialization. First, in genetically modified plants *E.coli* GUS can be regarded as safe for environment and consumers [11]. Second, in transgenic plants, overexpressed GUS is very stable and shows no toxicity in plant growth and development. In addition, detection of GUS does not require specialized equipment as it can easily be observed after staining with substrate such as 5-bromo-4-chloro-3-indolyl-β-d-glucuronic acid (X-GLUC). Thus, the GUS reporter system is a simple, reliable and cost-effective method to be used in transgenic studies [1], [12]. Despite these benefits of the *gusA* gene, false-positives are difficult to eliminate in some plants due to endogenous GUS activity [13]. Increasing the thermal stability of GUS through directed evolution *in vitro* could potentially eliminate false-positives and dramatically increase its veracity as a reporter.

The methodology of *in vitro* directed evolution has been approved in the laboratory [14], [15], [16]. Directed evolution has provided significant advances in many fields, such as for biocatalysis with industrially important enzymes [17], [18], [19], [20], [21], [22], plant improvement [23], [24], [25], [26], or in vaccines and pharmaceuticals [27], [28], [29].

Directed evolution *in vitro* via DNA shuffling, is an important method for generating proteins with improved functions. Stemmer introduced the method of *in vitro* DNA shuffling for the formation of recombinant genes from a set of parental genes [30], [31]. We have previously reported the directed evolution of a GUS variant, GUS-TR3337, which is significantly more resistant to high temperatures than wild-type enzyme, GUS-WT [32], [33]. Here, our objective was to test the utility of GUS-TR3337 as an improved reporter gene in a transgenic plant.

In order to study the activity of the thermostable GUS in plants, it was introduced into *Arabidopsis thaliana* by the floral dip method. The transgenic *Arabidopsis* plants containing the wild-type *gus-wt* gene (Genbank No. AF485783), lost most of the GUS activity within 5 min of exposure to 60°C. In contrast, *Arabidopsis* plants transformed with the *gus-tr3337* gene (GenBank No. DQ513152), retained the activity when heated upto 80°C for as long as 30 min. We confirm here that the mutant thermostable GUS gene, *gus-tr3337* is an improved reporter for transgenic research.

## Materials and Methods

### Chemicals, enzymes, plasmids and yeast strains

All reagents were purchased from Sigma Chemical Co. Ltd (St Louis, MO, USA) and Sinopharm Chemical Reagent Co. Ltd (Shanghai, China). Restriction enzymes were purchased from Promega (Madison, USA). High-fidelity DNA polymerase Pyrobest™ was purchased from Takara Co., Ltd (Dalian, China). DNA sequencing kit was purchased from Applied Systems Company (Foster City, CA, USA). QIAquick™ gel extraction kit was purchased from Qiagen (Stanford, CA, USA). X-GLUC and plasmid pBI121, containing the *gusA* gene, were purchased from Clontech (Palo Alto, CA, USA). *E. coli* strain DH5α used for amplification of plasmid DNA was purchased from Stratagene (La Jolla, CA, USA).

### Construction of plant expression vectors and plant transformation

The original *gus-wt* and mutant *gus-tr3337* genes were independently cloned in the binary vector pYF7716 that was constructed by our research team. This vector contains double-cauliflower mosaic virus 35S promoter, to direct transcription of the transgene as well as that of the intron-containing neomycin phosphotransferase II gene (*Km*). In addition, the vector carries the omega enhancer and the nopaline synthase terminator, that were fused to the tobacco SAR (scaffold attachment region) (Figure S1). The *gus-wt* and *gus-tr3337* constructs generated in this vector were introduced into *Agrobacterium tumefaciens* GV3101 by electroporation. *A. thaliana* cv. Columbia was transformed by the floral dip method as described previously [34]. The presence of the *gus* transgenes was confirmed via PCR amplification of the genes in *Arabidopsis* plants using the following specific primers pairs: GUS-WTF1 (5′-ATGTTACGTCCTGTAGAAACCCCAACC-3′) and GUS-WTR1 (5′-TCATTGTTTGCCTCCCTGCTGCGGTTTTTC-3′), for the *gus-wt* gene, and GUS-TRF1 (5′-TCACGCCGTACG TTATTGCCGGG-3′) and GUS-TRR1 (5′-TTGCTACCGCCAACGCGTAATATG-3′) for the *gus-tr3337* gene. The PCR conditions were: denaturation for 10 min at 94°C, followed by 30 cycles, each of 40 s at 94°C, 30 s at 58°C, 2 min at 72°C; and then extension for 10 min at 72°C. Standard DNA manipulation methods were used in plasmid construction and *E. coli* transformation [35].

### Histochemical analysis of GUS activity in transgenic *Arabidopsis* plants

For the analysis of expression of the *gus* transgenes, seeds of the trangenic *Arabidopsis* plants were sterilized with 1% (v/v) NaOCl solution and sown in pots containing a 1∶1∶1 mixture of peat moss/perlite/vermiculite. Pots were kept in a controlled-environment growth chamber at 22°C, programmed for a photoperiod of 16 h-day under cool white fluorescent light and 8 h-dark. High temperature treatments were performed on whole two-week old seedlings by exposure to temperatures of 70°C, 80°C, 30°C for different times (10 min, 20 min, 30, min). The heat treatment was given by placing a single seedling in a 0.6 mL capacity Eppendorf™ tube containing 150 µL ddH_2_0, and then holding the tube at the desired heating regime in a PCR System PTC-200, using the lid heating function to avoid the water evaporation. Then, the water was removed. Based on the apparent optimal temperature of β-glucuronidase activity tested in transgenic *Arabidopsis* plants, the plant samples were incubated at 45°C for transgenic *gus-wt Arabidopsis* samples or 60°C for transgenic *gus-tr3337 Arabidopsis* samples, with 1 mg/mL X-GLUC in a GUS activity buffer (50 mM sodium phosphate, pH 7.0; 1 mM EDTA; 0.1% Triton X-100; 0.5 mM K3[Fe(CN)6]; 0.5 mM K4[Fe(CN)6]·3H2O). Histochemical staining for GUS activity was performed essentially as described by Jefferson et al. [2] and Sudan et al. [36] for most experiments. The stained samples were immersed in 70% ethanol to remove chlorophyll. The ethanol solution was changed three times at 1-h intervals.

### GUS enzyme kinetics by fluorometric assay in transgenic *Arabidopsis* plants

The GUS activity in plant samples was measured by a fluorometric assay using DyNA Quant 200™ Fluorometer (Hoefer Pharmacia Biotech, San Francisco, CA, USA) as described by Martin et al. [37] and Fior et al. [38] for most experiments. Standards were prepared with different concentrations of 4-methylumbelliferone sodium salt (4-MU, Sigma Chemical Co. Ltd., No. M1508): 1 µM, 1 mM and 100 mM, in 0.2 M sodium carbonate. 4-Methylumbelliferyl-β-D-glucuronide (4-MUG, Sigma Chemical Co. Ltd., No. M5664), 2 mM, was added to sample as substrate. GUS extraction buffer (50 mM sodium phosphate, pH 7.0; 10 mM β-mercaptoethanol; 10 mM EDTA and 0.1% Triton X-100) was used as reaction medium.

Extracts of whole three-week-old seedlings in GUS extraction buffer were used for the analysis of enzyme kinetics. Seedlings were frozen by immersion in liquid nitrogen and ground with a pestle and mortar in the presence of liquid nitrogen. GUS extraction buffer, 500 µL, was added and the plant material was vigorously ground to a finely pulverized powder. The extract thus obtained was centrifuged at 8,000× *g* for 1 min at 4°C. The supernatant was recovered and diluted one-hundred fold with GUS extraction buffer in order to decrease the effect of any potential indigenous GUS inhibitors [39]. The diluted extract, 10 µL was added to 4-MUG, 90 µL (2 mM), to initiate the reaction. After 1 h the reaction was stopped by the addition of 900 µL of 0.2 M Na_2_CO_3_, and fluorescence was measured using a DyNA Quant 200™ spectrofluorometer.

The optimal pH for the activity of enzymes GUS-WT and GUS-TR3337 from transgenic *Arabidopsis* plants was determined at 37°C at every half point between pH values 2.5 and 13.0. The activity of both enzymes from transgenic *Arabidopsis* plants was measured at 5-degree intervals between 25°C and 90°C to determine the optimum temperature at which maximum activity was retained. The thermostability of the two enzymes from transgenic *Arabidopsis* plants was determined by measuring the residual activity at 37°C after subjecting the samples to temperatures of 60°C, 70°C, 80°C and 90°C for different times (10 min, 20 min and 30 min), followed by chilling on ice. All reactions were performed in triplicate. Kinetic parameters were determined as described in previous reports [32], [40], [41].

## Results

### Over-expression of *gus-wt* and *gus-tr3337* in *Arabidopsis*

The cloned *gus-wt* or *gus-tr3337* genes under the control of the CaMV 35S promoter were independently introduced into *Arabidopsis* (Figure S1). The transgenic *Arabidopsis* plants thus recovered showed high levels of expression of the two genes (Figure S2). There were no noticeable differences in the morphology or growth among the above transgenic plants and the parental *Arabidopsis* plants (data not shown).

### Thermostability of the mutant GUS-TR3337 in transgenic *Arabidopsis* plant

**Figure 1:**
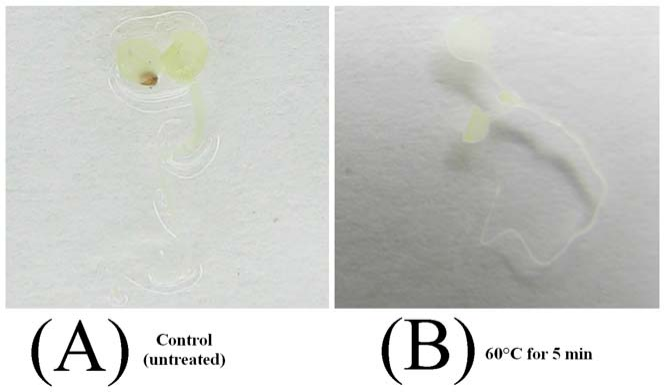
Histochemical staining of GUS activity in wild-type *Arabidopsis* seedlings. Seedlings were subjected to heat treatment and then stained at with X-GLUC 37°C overnight. A: Control (untreated); B: 60°C for 5 min.

**Figure 2:**
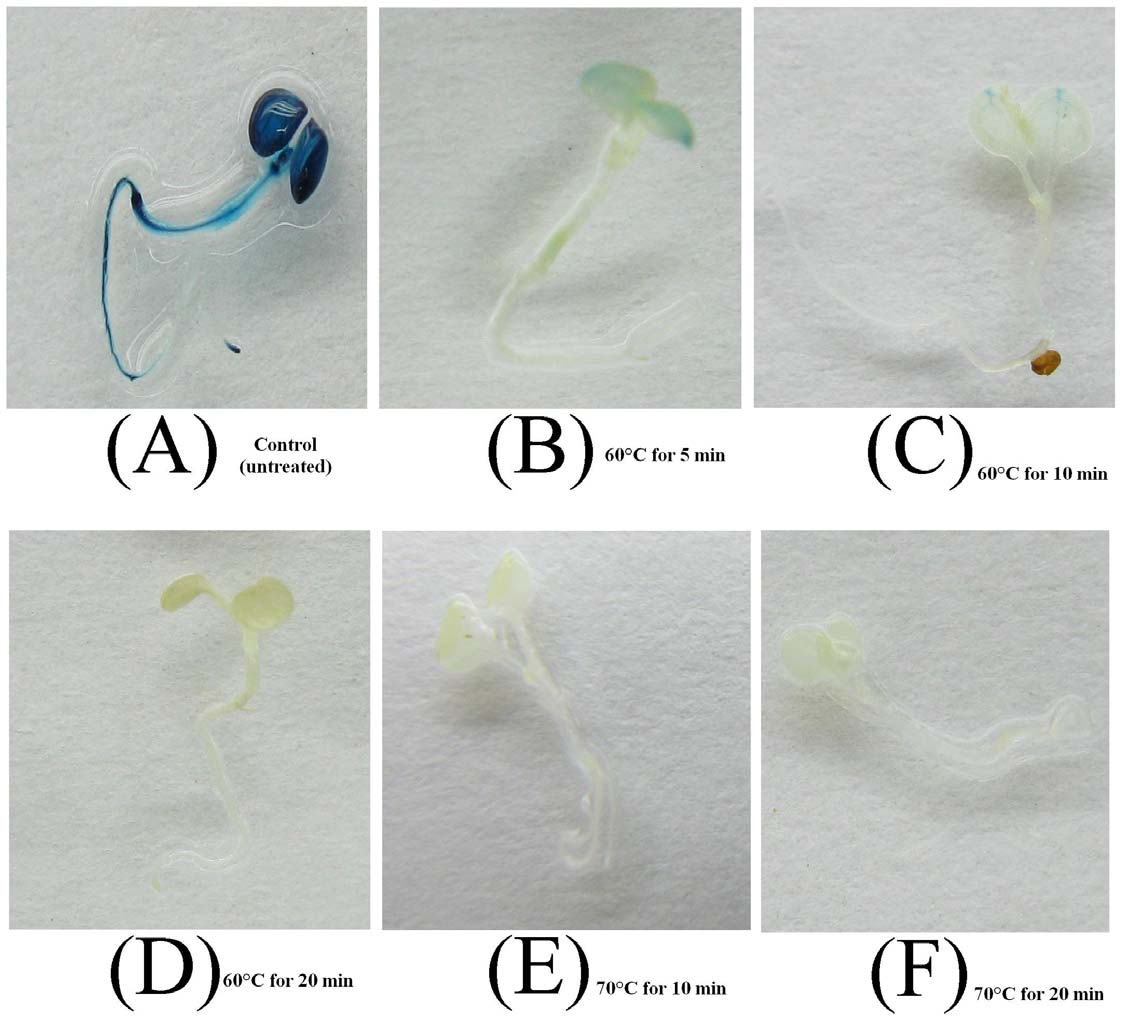
Histochemical staining of GUS activity in transgenic *Arabidopsis* seedlings hosting the *gus-wt* gene. Seedlings were subjected to various heat treatments and then stained at with X-GLUC 45°C overnight. (A) Control (untreated); (B) 60°C for 5 min; (C) 60°C for 10 min; (D) 60°C for 20 min; (E) 70°C for 10 min; (F) 70°C for 20 min.

**Figure 3:**
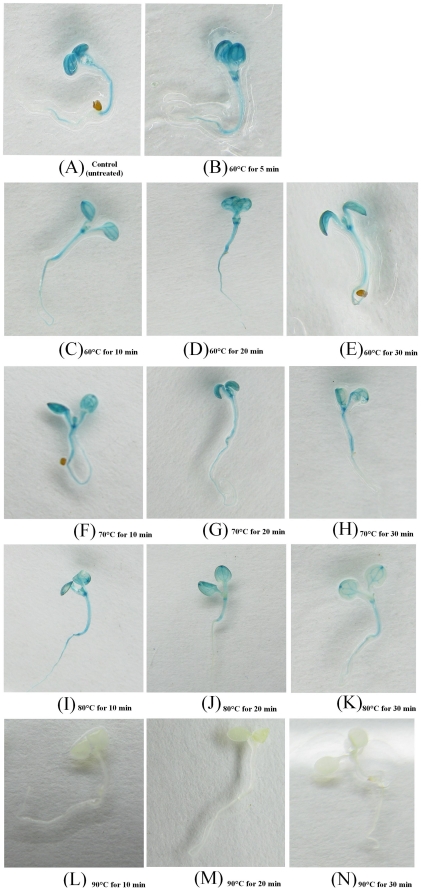
Histochemical staining of GUS activity in transgenic *Arabidopsis* seedlings hosting the *gus-tr3337* gene. Seedlings were subjected to various heat treatments and then stained at with X-GLUC 60°C overnight. (A) Control (untreated); (B) 60°C for 5 min; (C–E) 60°C for 10 min, 20 min or 30 min respectively; (F–H) 70°C for 10 min, 20 min or 30 min, respectively; (I–K) 80°C for 10 min, 20 min or 30 min, respectively; (L–N) 90°C for 10 min, 20 min or 30 min, respectively.

High temperature treatments were performed with two-week-old whole seedlings to evaluate the thermostability of the mutant GUS-TR3337 in transgenic *Arabidopsis* plants. Seedlings of wild-type *Arabidopsis* could not been stained by X-GLUC when they were not or subjected to 60°C for 5 min (Figure 1). Seedlings containing the wild-type *gus-wt* gene or the mutant *gus-tr3337* gene exhibited stable GUS activity when they were not subjected to high temperature treatments (Figures 2A and 3A). The seedlings expressing the *gus-wt* gene were stained weakly by X-GLUC after being subjected to 60°C for 5 min or 10 min (Figures 2B and 2C) and the enzyme appeared to be inactivated by treatments for longer duration or at higher temperature (Figures 2D–F).

On the opposite, seedlings expressing the mutant *gus-tr3337* gene exhibited stable GUS activity when treated at 60°C and 70°C for 10 min, 20 min or 30 min (Figures 3C–E and F–H), and even at 80°C for 10 min, 20 min or 30 min (Figures 3I–K). However, treatment at 90°C for 10 min or longer appeared to inactivate the GUS-TR3337 enzyme (Figures 3H–I).

### Characterization of the GUS-TR3337 activity in transgenic *Arabidopsis* plants

**Figure 4:**
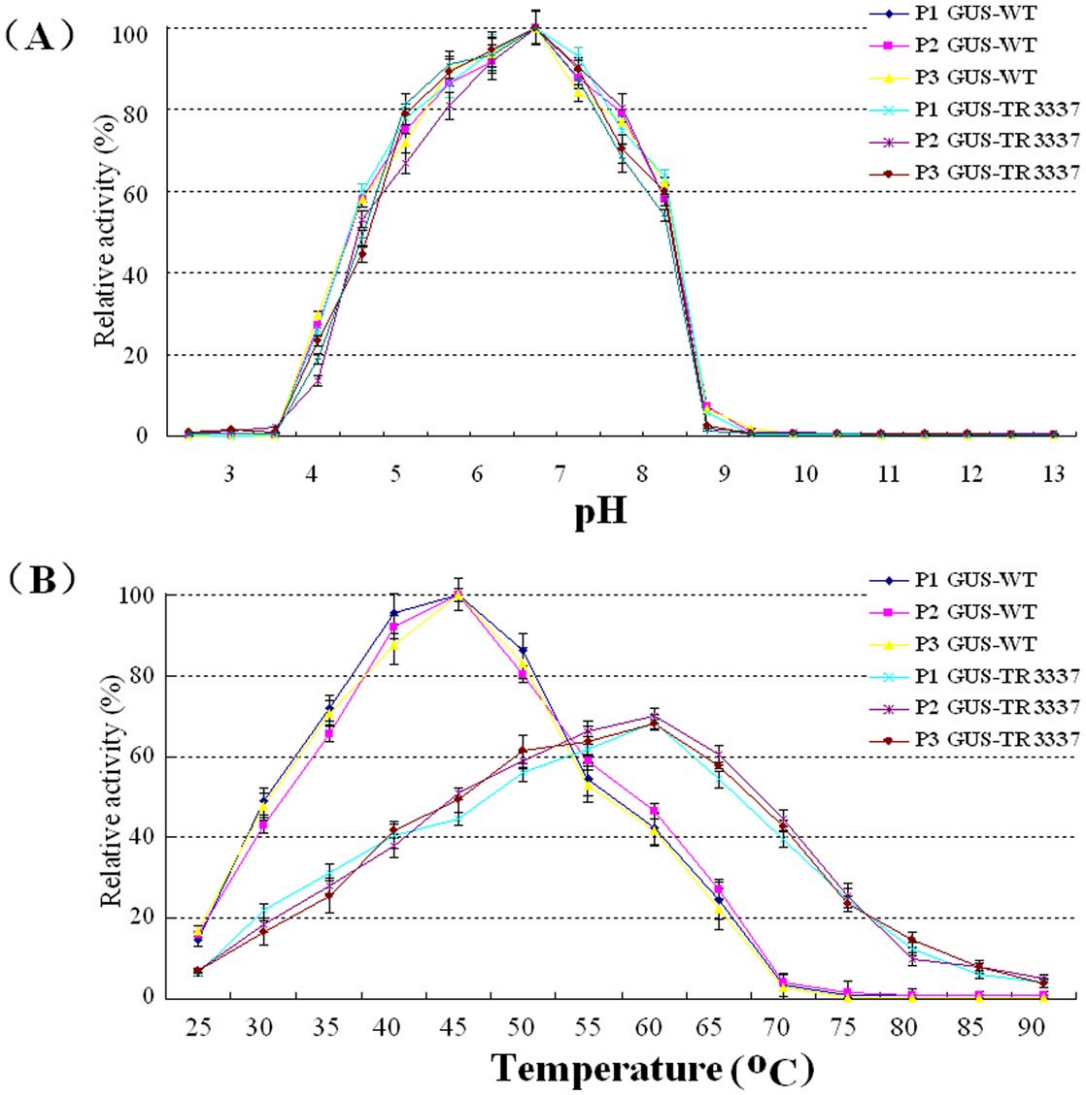
pH- and temperature-dependence of GUS activity in transgenic *Arabidopsis* plants hosting the *gus-tr3337* gene or the *gus-wt* gene. P1. P2. P3 are triplicates. (A) Enzymatic activity in seedling extracts was measured in buffers at the indicated pH; (B) Enzymatic activity in seedling extracts was measured in phosphate buffer at pH 7.0 at the indicated temperatures. Values are mean ± S.D. of triplicates.

Three-week-old transgenic *Arabidopsis* seedlings containing the mutant *gus-tr3337* gene or the wild-type *gus-wt* gene were used to characterize the GUS-TR3337 and GUS-WT enzymes in seedling extracts. Three representative seedlings each (P1, P2 and P3) expressing either of transgenes were tested using 4-MUG as substrate at 37°C. A single activity peak was found at pH 6.5 in all six transgenic *Arabidopsis* lines expressing *gus-tr3337* or *gus-wt* gene (Figure 4A).

The apparent optimal temperature of GUS activity tested at pH 7.0 in extracts of transgenic *Arabidopsis* plants over-expressing *gus-wt* gene was 45°C, while that of transgenic *Arabidopsis* plants over-expressing *gus-tr3337* gene was 60°C (Figure 4B). The GUS-WT activity in transgenic *Arabidopsis* lines at 45°C was about 30% higher than that at 35°C. The GUS-TR3337 activity in transgenic *Arabidopsis* lines at 60°C was >2 times of its activity at 35°C.

**Figure 5:**
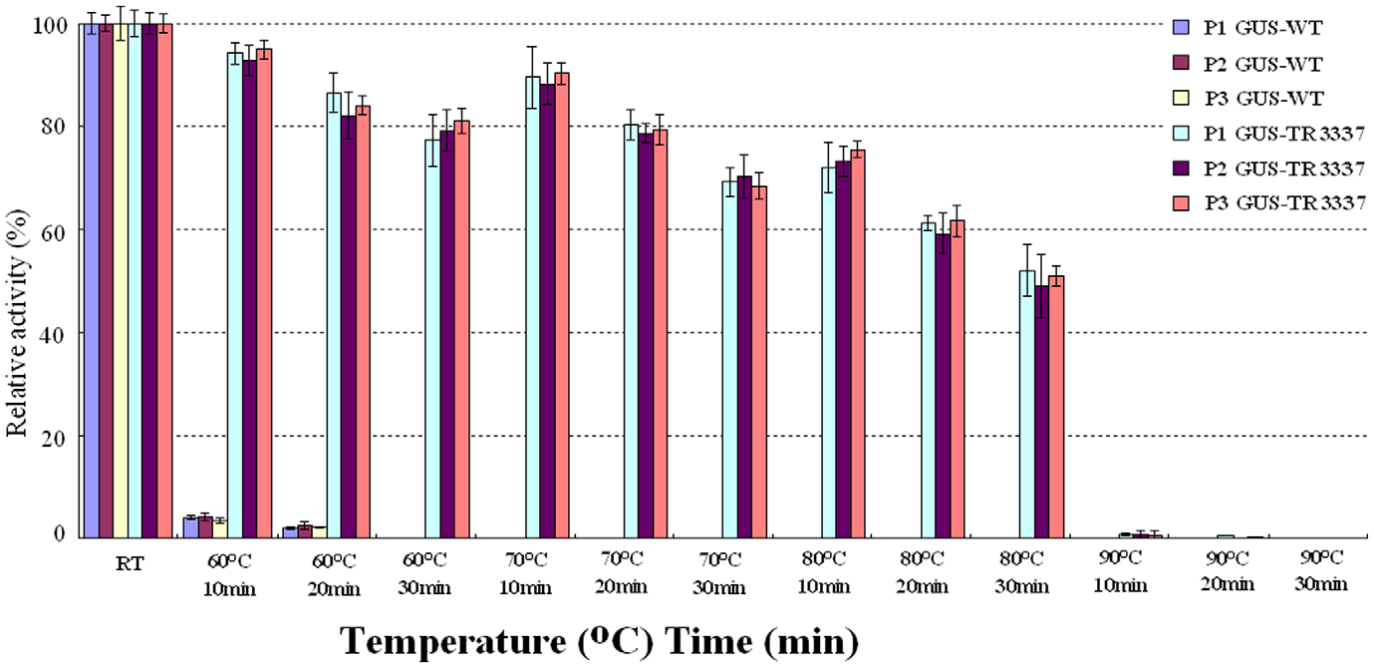
Thermostability of GUS in transgenic *Arabidopsis* plants. P1-, P2-, P3-GUS-TR3337: transgenic *Arabidopsis* plant lines hosting the mutant *gus-tr3337* gene; P1-, P2-, P3-GUS-WT: transgenic *Arabidopsis* plant lines hosting the wild-type *gus-wt* gene. Seedlings were subjected to heat treatments at the indicated temperature and duration and the residual GUS activity was measured in the plant extracts at 37°C. Values are mean ± S.D. of triplicates.

The GUS-WT activity in the extracts of transgenic *Arabidopsis* decreased significantly after being subjected to 60°C for 10 min, and was undetectable after treatment at 70°C or higher temperatures for 10 min or longer (Figure. 5). In contrast, GUS-TR3337 activity in the transgenic *Arabidopsis* plants was much more resistant to heat treatment. Nearly 90% and 70% of GUS activity was retained when plants were heated at 70°C for 10 min or 30 min, and about 70% and 50% was retained when heated at 80°C for 10 min or 30 min.

Similarly, when heated at 70°C for 10 min or 30 min, they retained nearly 90% and 70% of this activity, respectively; and when heated at 80°C for 10 min or 30 min, retained about 70% and 50%, of this activity, respectively. The GUS-TR3337 activity decreased significantly following at heat treatment 90°C for 10 min and was undetectable if subjected to 90°C or higher temperatures for 30 min or longer (Figure 5).

## Discussion

Various reporter genes encoding GFP, GAL, LUC, GUS, or oxalate oxidase (OxO) have been used in transgenic plant research or crop development, and have been assessed for the efficiency, scientific application and commercialization. Many of these genes have specific limitations or have not been sufficiently tested to merit their widespread use [1]. There are some reports on improved reporter genes that allow easy detection or a wide range of applications and that are generated by directing their evolution in the laboratory. Crameri and his colleagues used DNA shuffling to obtain GFP variants with 40 times increased fluorescence intensity [42]. A mutant GFP with stronger fluorescence intensity was selected by directed evolution in conjunction with a functional salvage screen [43].

The GUS enzyme encoded by *gusA* gene of *E. coli* is still to date the most widely used reporter gene in plants. Some limitations of using GUS as a reporter are the activity assay that is destructive to the plants and the false positive events due to endogenous GUS or GUS-like enzymes activity in some plants [44]. Hu et al. reported that there were intrinsic GUS-like activities in some seed plants [13]. Some novel GUS or GUS-like genes were also isolated and characterized from plants, such as *Scutellaria baicalensis Georgi* [45]. GUS-like enzymes were known to be present in microorganisms [2], [3], [46], [47], animals [48], [49] and plants [11], [13], [36], [39], [50], such as *Scutellaria baicalensis* [51], rye [52], and maize [53]. Sudan and his colleagues demonstrated that GUS is ubiquitously present in plants [36]. Over the past three decades, numerous reports have illustrated that there was GUS background activity in different plant species, including some model plants, such as *Arabidopsis thaliana*, *Oryza sativa*, *Nicotiana tabacum* and *Zea mays* [36], [38], [54], [55], [56].

Therefore, efforts have been focused on the suppression of the background activity [57], [58], [59]. As reported by Kosugi et al., the addition of methanol to 20% of the final volume during GUS staining, or during fluorimetric assays, eliminates the endogenous activities found in many plants [59]. The methanol appears to have no effect on the bacterial GUS encoded by the *gusA* from *E. coli*. However, methanol is not always effective in suppressing the endogenous GUS activities, and moreover, it is harmful to the experimentalists.

The *E. coli* GUS system is a good model system for the study of directed evolution *in vitro*. Matsumura et al. have directed the evolution of a GUS variant that is significantly more resistant to both glutaraldehyde and formaldehyde than the wild-type enzyme [40]. This GUS variant could resolve the problem of loss of GUS activity in transgenic plants during tissue fixation by glutaraldehyde or formaldehyde. After four cycles of screening the best variant was more active than the wild-type enzyme, and retained function at 70°C, whereas the wild-type enzyme lost activity at 65°C [41]. DNA shuffling (sexual recombination) and a histochemical screen have also been used to direct the evolution of *E. coli* GUS variants with improved β-glucuronidase activity [60]. The same model evolutionary system was employed to test the efficiency of several other techniques [61]. One of the GUS variants was obtained by screening for increased p-nitrophenyl-β-d-xylopyranoside (pNP-xyl) activity [62]. Although there have been a few studies on directed evolution of GUS to obtain an improved reporter, none of them have been in transgenic plants.

We obtained a temperature resistant *gusA* variant *gus-tr3337* (GenBank No. DQ513151) by employing a high throughput system for directed evolution *in vitro* [32], [33]. The mutant enzyme maintained its activity even when the nitrocellulose filter containing the variant colony was heated at 100°C for 30 min. Sequencing of the *gus-tr3337* revealed six mutations: Q493R, T509A, M532T, N550S, G559S and N566S that are present in GUS-TR3337, indicating that these were the key amino acids needed to confer its high thermostability. The purified GUS-TR3337 protein retained most of its activity when heated at 80°C for 10 min, while the wild-type GUS protein lost its activity completely after 10 min at 70°C. With pNPG as a substrate, the purified GUS-TR3337 protein retained 75% or 48% of its activity when heated at 80°C for 10 min or 30 min, respectively [33]. In the present study, with 4-MUG as substrate, transgenic *Arabidopsis* plants hosting the *gus-tr3337* gene retained about 70% and 50% of GUS activity when heated at 80°C for 10 min or 30 min, respectively. With pNPG as substrate, a single activity peak had been recorded at pH 6.5 at 37°C with the purified GUS-WT and GUS-TR3337 enzymes [33]. Similar results were obtained in the present study on GUS enzymes in extracts from transgenic *Arabidopsis* plants overexpressing the *gus-tr3337* gene and the *gus-wt* gene. The apparent optimal temperature for GUS-WT and GUS-TR3337 tested at pH 6.5 was 45°C and 65°C, respectively with pNPG as substrate; while temperature optima for the two enzymes expressed in *Arabidopsis* plants, as tested with 4-MUG as a substrate at pH 7.0 was 45°C and 60°C respectively. Thus, the apparent temperature optimum of GUS-TR3337 expressed in transgenic plants was 5°C lower than that of purified enzyme, under the conditions of the assays. We also repeated the characterization (determination of the optimal pH, temperature and of residual activity after heat treatment) of the purified enzymes of GUS-TR3337 and GUS-WT by comparing pNPG and MUG as substrates and found that the two substrates yield similar results (Figures S3 and S4).

Although the GUS system is the most popular transgenic reporter gene for plants [1], there were some limitations of the use GUS system as a reporter. Other potential problems with GUS, as discussed by Taylor [44], include its false positive, particularly in those plants with endogenous high endogenous GUS or GUS like activity that interferes with the histochemical detection of transgenic GUS. In the present study, transgenic *Arabidopsis* plants with the *gus-tr3337* gene (GenBank No. DQ513152) retained GUS enzyme activity in plant extracts as well *in vivo* even after being heated at 80°C for up to 30 min, while plants with the *gus-wt* gene lost both *in vitro* and *in vivo* enzyme activity almost completely after 10 min at 60°C. Based on the staining with 1 mg/mL X-GLUC the apparent temperature optimum for GUS-TR3337 activity was found to be 60°C. The transgenic *Arabidopsis* plant hosting the *gus-tr3337* gene could be easily staining with X-GLUC after heated at 80°C for 10 min to 30 min. The thermostability of GUS-TR3337 can be exploited to minimize the recovery of false positives. Some limitations of GUS as a reporter could be overcome by using this thermostable GUS gene (*gus-tr3337*). Staining with X-GLUC after heat treatment at 70°C for 30 min or at 80° for 10–30 min will allow to distinguish the GUS activity associated to the transgenic gus-tr3337 expression from the endogenous one, responsible of false positives, in the plants where the endogenous Gus or GUS-like activity is not thermostable. In conclusion, the use of *gus-tr3337* transgene can enhance the precision, sensitivity, and reliability of *gus* gene as a reporter.

## Supporting Information

Figure S1 **Schematic diagram of the vectors used in this study.** The original (*gus-wt*) and mutant (*gus-tr3337*) genes was cloned into the binary vector pYF7716, at the *Bam* HI and *Sac* I restriction enzyme sites, under the control of double CaMV 35S promoter (D35S). For steady transmission of *gus-wt* and mutant *gus-tr3337*, two scaffold attachment regions (SAR) were fused upstream of the D35S promoter and downstream of the Nos-Terminator (Nos-T).
(BMP)

Figure S2 **Identification of T_1_ generation of transgenic plants.** (A) Screening of T_1_ generation seeds on kanamycin containing plates; (B) Screening of T_1_ transgenic seedlings after staining for GUS activity.
(TIF)

Figure S3 **pH- and temperature-dependence of GUS activity in purified protein with 4-MUG as a substrate.** (A) Enzymatic activity was measured at the indicated pH; (B) Enzymatic activity was measured at the indicated temperatures. Values are mean ± S.D. of triplicates.
(BMP)

Figure S4 **Thermostability of GUS in purified protein with 4-MUG as a substrate.** Proteins were subjected to heat treatments at the indicated temperature and duration and the residual GUS activity was measured at 37°C. Values are mean ± S.D. of triplicates.
(BMP)
