# Supplementary figures and images for: A Thermostable β-Glucuronidase Obtained by Directed Evolution as a Reporter Gene in Transgenic Plants
Source: PLoS One. 2011 Nov 9;6(11):e26773. doi: 10.1371/journal.pone.0026773 (PMC3212524; doi:10.1371/journal.pone.0026773)

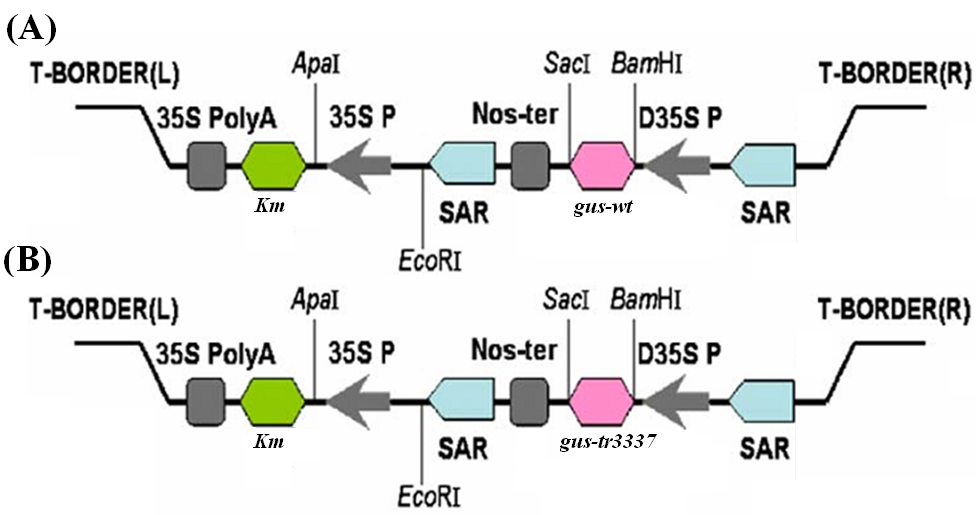

Supplement: Figure S1 — Schematic diagram of the vectors used in this study. The original (gus-wt) and mutant (gus-tr3337) genes was cloned into the binary vector pYF7716, at the Bam HI and Sac I restriction enzyme sites, under the control of double CaMV 35S promoter (D35S). For steady transmission of gus-wt and mutant gus-tr3337, two scaffold attachment regions (SAR) were fused upstream of the D35S promoter and downstream of the Nos-Terminator (Nos-T). (BMP) [file pone.0026773.s001.bmp]

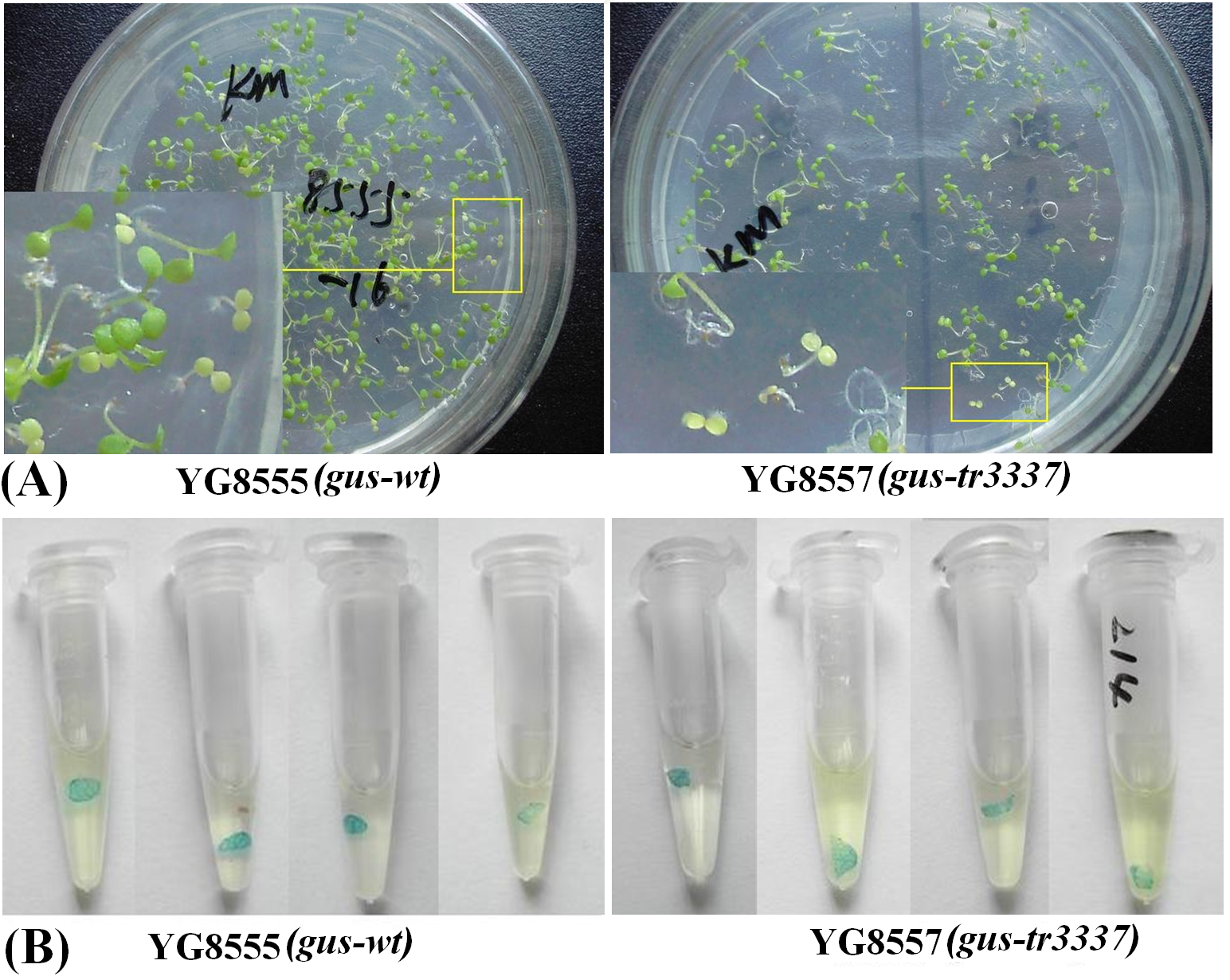

Supplement: Figure S2 — Identification of T1 generation of transgenic plants. (A) Screening of T1 generation seeds on kanamycin containing plates; (B) Screening of T1 transgenic seedlings after staining for GUS activity. (TIF) [file pone.0026773.s002.tif]

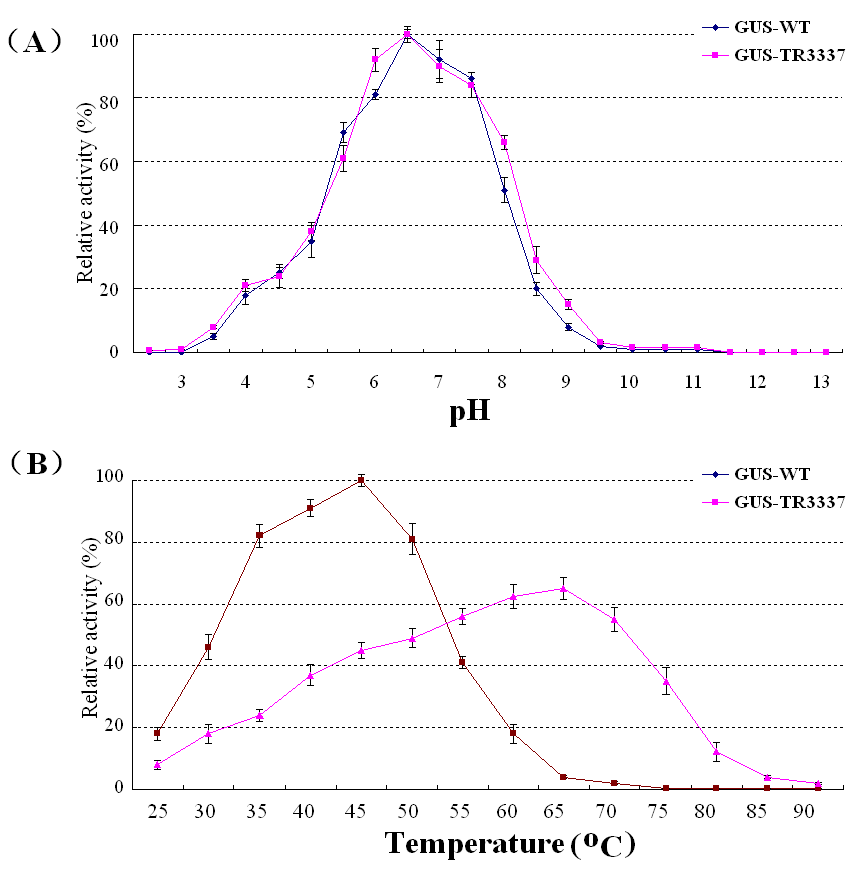

Supplement: Figure S3 — pH- and temperature-dependence of GUS activity in purified protein with 4-MUG as a substrate. (A) Enzymatic activity was measured at the indicated pH; (B) Enzymatic activity was measured at the indicated temperatures. Values are mean ± S.D. of triplicates. (BMP) [file pone.0026773.s003.bmp]

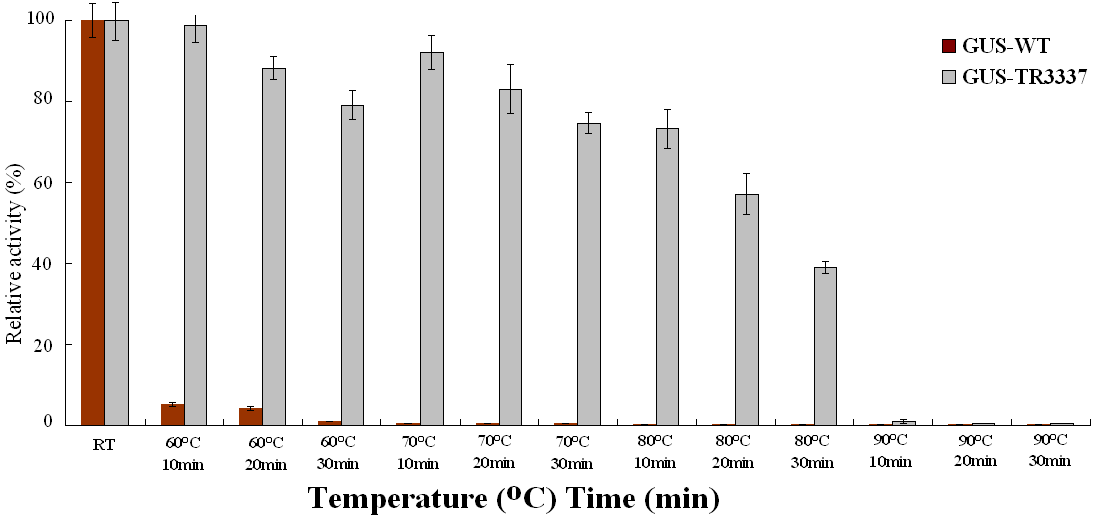

Supplement: Figure S4 — Thermostability of GUS in purified protein with 4-MUG as a substrate. Proteins were subjected to heat treatments at the indicated temperature and duration and the residual GUS activity was measured at 37°C. Values are mean ± S.D. of triplicates. (BMP) [file pone.0026773.s004.bmp]
